# Adaptive Bilateral Texture Filter for Image Smoothing

**DOI:** 10.3389/fnbot.2022.729924

**Published:** 2022-06-27

**Authors:** Huiqin Xu, Zhongrong Zhang, Yin Gao, Haizhong Liu, Feng Xie, Jun Li

**Affiliations:** ^1^School of Mathematics and Physics, Lanzhou Jiaotong University, Lanzhou, China; ^2^Fujian Science & Technology Innovation Laboratory for Optoelectronic Information of China, Fuzhou, China; ^3^Quanzhou Institute of Equipment Manufacturing, Chinese Academy of Sciences (CAS), Quanzhou, China; ^4^Institute of Automation and Communication Magdeburg, Magdeburg, Germany

**Keywords:** image smoothing, bilateral filter, structure measurement, adaptive spatial kernel, Fourier approximation

## Abstract

The biggest challenge of texture filtering is to smooth the strong gradient textures while maintaining the weak structures, which is difficult to achieve with current methods. Based on this, we propose a scale-adaptive texture filtering algorithm in this paper. First, the four-directional detection with gradient information is proposed for structure measurement. Second, the spatial kernel scale for each pixel is obtained based on the structure information; the larger spatial kernel is for pixels in textural regions to enhance the smoothness, while the smaller spatial kernel is for pixels on structures to maintain the edges. Finally, we adopt the Fourier approximation of range kernel, which reduces computational complexity without compromising the filtering visual quality. By subjective and objective analysis, our method outperforms the previous methods in eliminating the textures while preserving main structures and also has advantages in structure similarity and visual perception quality.

## Introduction

Natural images usually have complicated textures, which makes it difficult to understand the main information of the image without texture removal. Structure-preserving texture smoothing is an important issue in computer vision and digital image processing for image cognition. It attempts to eliminate the meaningless textures while preserving dominant structure as well as possible, which has a wide range of applications, such as tone mapping (Jia and Zhang, 2019), detail enhancement (Fei et al., 2017), image abstraction (Winnemöller et al., 2006), and so on. For structure-preserving texture filtering, the first is to detect pixels near structure edges and then preserve structures while eliminating textures. Therefore, texture filtering plays an essential role in many image preprocessing applications.

The early methods utilized were in contrast to pixel intensity for texture measurement (Tomasi and Manduchi, 1998; Farbman et al., 2008; Xu et al., 2011). Such methods can remove fine details but perform poorly when directly eliminating high-contrast and complicated textures in the image. Subsequently, some more comprehensive texture measurement methods have been proposed, such as local extrema (Subr et al., 2009), region covariance (Karacan et al., 2013), and relative total variation (Xu et al., 2012), these methods can smooth out the textures but also cause blurring of the small structure edges. Further, many scholars have improved texture measurement methods to generate the guidance image in the joint bilateral filter. For instance, the guidance image is calculated through patch shift for each pixel (Cho et al., 2014). Similarly, joint bilateral filtering was also employed in Jeon et al. (2016), Song et al. (2018) and Xu and Wang (2019), where they use an adaptive kernel scale to generate a smoothed image as guidance. These methods perform better because they proposed to use small window sizes near structures and large window sizes in the textures region.

On the other hand, some methods were introduced by adaptively adjusting the spatial kernel scale or range kernel scale of the bilateral filter. For instance, the size of the range kernel is changed at each pixel (Gavaskar and Chaudhury, 2018), where the polynomials are adopted to approximate histograms for accelerations of adaptive bilateral filtering. In addition, the width of the spatial kernel is adapted by relying on local gradient information (Ghosh et al., 2019), which can obtain structure-preserving smoothing results. This paper modifies the structure measure used in Ghosh et al. (2019) to implement the superior performance of texture removal.

In recent years, deep learning algorithms have been introduced to the area of edge-preserving texture filtering. Earlier work included deep edge-aware filtering proposed by Xu et al. (2015), which constructs a unified neural network architecture in the gradient domain. Chen et al. (2017) and Lu et al. (2018) both trained fully supervised Convolutional Neural Networks for texture smoothing. Since the above methods require several image pairs that are not readily available to train the model, the semi-supervised method (Gao et al., 2020) and unsupervised method (Zhu et al., 2017) are proposed to avoid the collection of annotated training examples.

In this paper, we present a scale-adaptive texture smoothing algorithm based on the traditional bilateral filtering framework, which smooths multi-scale textures by adjusting the scale of the spatial kernel at each pixel. First, we employ gradient information along with the four-directional structure detection to identify the structures from coarse textures. Second, the spatial kernel size for each pixel is estimated depending on the structure measure. Finally, we use the Fourier approximation of the Gaussian range kernel to accelerate the bilateral filtering for texture removal, where the computational complexity does not change with the spatial kernel size. The experimental results show that our method can effectively achieve the outstanding capability of structure-preserving smoothing results. The main contributions of this paper are as follows:

- We propose a four-directional structure detection based on gradient information, which uses the gradient information in the pixel neighborhood to more accurately extract structures from images containing complicated textures.
- We propose a mapping rule to determine the spatial kernel scale of each pixel, which can adaptively adjust scale size via structure information. The pixels in the vicinity of the structure edges adopt smaller spatial kernel scales and the pixels in textural regions adopt larger spatial kernel scales.
- The approximation algorithm of adaptive bilateral filtering is presented for texture removal. This strategy claims that the complexity of texture filtering does not lie on the scale of the spatial kernel.

In the following section of this paper, the related work is described in Section Related Work, our proposed method is discussed in detail in Section Our Method, experimental analysis is discussed in Section Experiments and Results, the applications of our algorithm are presented in Section Applications, and a conclusion is introduced in Section Conclusion.

## Related Work

The research of texture filtering has received a lot of attention in the past several decades.

Traditional texture filtering algorithms include local weighted averaging and global optimization. Bilateral filtering (BF) (Tomasi and Manduchi, 1998), guided filter (He et al., 2013), and anisotropic diffusion (Perona and Malik, 1990) are all typical local weighted averaging methods. As one of the classic non-linear filters, BF combines the spatial kernel and the range kernel for noise removal. Algorithms based on global optimization mainly include the total variation (TV) model (Rudin et al., 1992), weighted least squares (WLS) (Farbman et al., 2008), and *L*_0_ gradient minimization (Xu et al., 2011); these methods optimize the global framework that relies on gradient information, which can overcome some limitations of local filters such as halo artifacts and gradient reversals, but these optimization-based methods need to solve a complex linear model which is time-consuming and cannot remove high-contrast noise well. Subsequently, some edge-preserving models have been proposed to optimize the global framework, for example, Huang et al. (2018) took advantage of global optimization together with local filtering to enhance the smoothness. To improve smoothing quality and processing speed based on WLS, Liu et al. (2017) proposed semi-global weighted least squares which solve a sequence of subsystems iteratively, and Liu W., et al. (2020) achieved high speed through Fourier transform and inverse transform. However, these traditional texture filters cannot effectively distinguish prominent structures from complex details and completely smooth out the textures in images with complex backgrounds.

Some new models have been proposed for extracting the salient structure from the input images, which make use of texture characteristics instead of gradient information to identify regular or random textures. For example, Subr et al. (2009) decomposed the structures and textures through local extrema, and they defined textures as the oscillations between local minima and maxima and calculated the average of the extremal envelopes to smooth out the textures. Karacan et al. (2013) proposed a patch-based region covariance that uses first-order and second-order feature statistics to extract structures from different types of textures; however, structures that have similar statistical properties to textures may be incorrectly smoothed out, which tend to overly blur the structures of the images. Lee et al. (2017) proposed an interval gradient operator for structure-preserving image smoothing. On the other hand, Xu et al. (2012) observed that the inherent variation in a window that includes structures is generally greater than that in a window containing textures, so they propose a relative total variation (RTV) to capture the structures and textures characteristics of the images. Subsequently, Zhao et al. (2019) proposed an activity-driven LAD-RTV for texture removal. However, these methods may incorrectly regard small structures as texture because of the overlapping area between adjacent windows. The complex textures cannot be completely smoothed out when the smoothing window size is too small, while an excessively smoothed image may be produced, and when the size is too large, it is difficult to find a suitable window size to achieve the balance between preserving main structures and removing unimportant textures.

To address the limitations of not smoothing out complicated textures and structure edges blurring, filtering methods based on structure-aware have been proposed to achieve high-smoothing quality. That is, the smoothing scale for a pixel is adaptively varied from pixel to pixel. These methods obtain smoothing results through joint bilateral filtering, in which the guidance image calculated by adaptive kernel scale is a particularly important process. Jeon et al. (2016) propose a scale-adaptive texture filtering based on patch-based statistics, and the optimal smoothing scale of each pixel is estimated according to the directional relative total variation (dRTV) measurement. Song and Xiao (2017) used patches of two scales to represent pixels by calculating the directional anisotropic structure measurement (DASM) on each pixel; the smaller patches are adopted for pixels at the structures and the larger patches are adopted for pixels in texture regions. Subsequently, Song et al. (2019) utilized directional anisotropic structure measure (DASM) to replace dRTV in Jeon et al. (2016), then evaluate the exact smoothing scale relying on four-direction statistics of DASM value. With regard to texture measurement windows, Xu and Wang (2018) adopted long and narrow small windows for texture measurement because the structure edges are not always parallel to the axes. Furthermore, Liu Y., et al. (2020) proposed texture filtering based on the local histogram operator, which uses the difference in color distribution to distinguish structures from textures, and then they determined the width of the range kernel. The above methods perform well in preserving structure while smoothing out textures. However, the multiple iterations of joint bilateral filtering may cause blurred structure and color cast.

Recently, deep learning has made significant progress in the field of image texture smoothing (Chen et al., 2017; Kim et al., 2018; Gao et al., 2020). Kim et al. (2018) designed a new framework for structure-texture decomposition, and they replaced the total variation prior with a network and plug deep variational priors into an iterative smoothing process. Gao et al. (2020) presented a semi-supervised algorithm relying on Generative Adversarial Networks (GANs) for structure-preserving smoothing, which designs different loss functions for both labeled and unlabeled datasets. However, in neural network training, their target outputs are usually generated by the existing smoothing methods.

## Our Method

**Figure 1:**
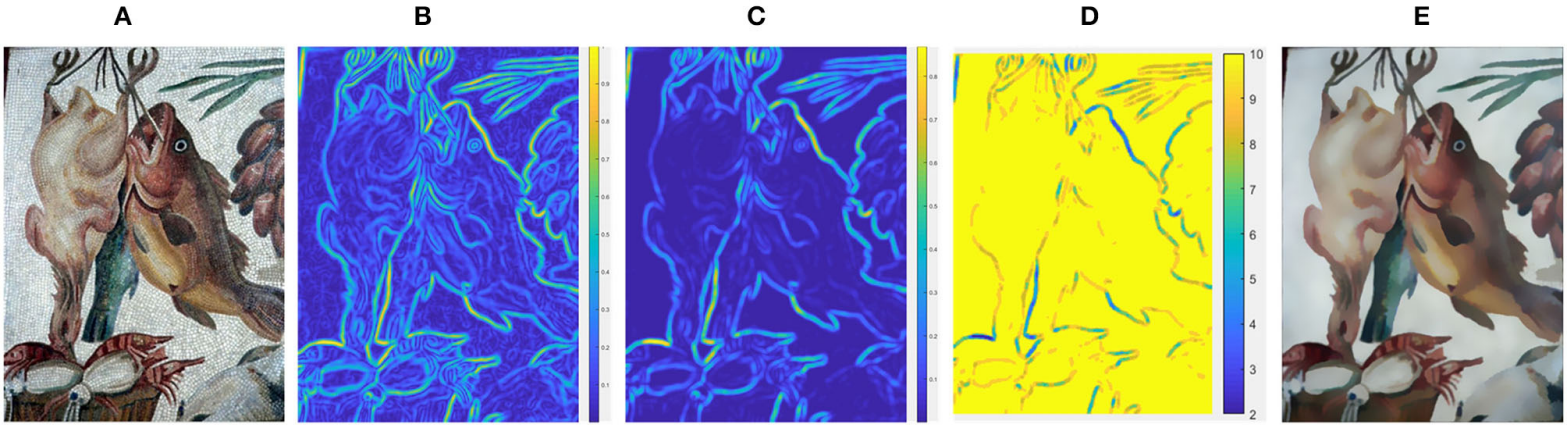
The process of texture filtering for the input image. **(A)** Input image; **(B)** Gradient map; **(C)** Structure map; **(D)** Scale map; **(E)** Smoothing result. Reproduced with permission from Hyunjoon Lee, Junho Jeon, Junho Kim and Seungyong Lee, available at https://sci-hub.wf/10.1111/cgf.12875.

Classic bilateral filtering makes use of spatial kernel together with range kernel, which not only notices the distance between pixels but also pays attention to the similarity of the intensity of pixels. Based on this, we propose a scale adaptive bilateral filtering that allows the scale of the spatial kernel to adjust at each pixel. Figure 1 shows the entire process of texture filtering of our method.

### Structure-Preserving Bilateral Filtering

Considering the general form of bilateral filtering Tomasi and Manduchi, 1998, for the input image *f*, the output result is obtained by scale adaptive bilateral filtering, written as:


(1) $$\begin{array}{l} u\left(p\right)=\frac{\sum_{q\in \Omega_{p}}w\left(q-p\right)\varphi \left(f\left(q\right)-f\left(p\right)\right)f\left(q\right)}{\sum_{q\in \Omega_{p}}w\left(q-p\right)\varphi \left(f\left(q\right)-f\left(p\right)\right)}, \end{array}$$

where *u* (*p*) is the output value at the pixel *p*, and *w* (*l*) and φ (*t*) represent the spatial kernel and the range kernel, respectively. We use a box function for the spatial kernel in this paper, that is, the window Ω_*p*_ of the spatial kernel centered at the pixel *p*. We assume that *W*_*p*_ represents the scale of the spatial kernel at the pixel *p*, then Ω_*p*_ can be expressed as $|\Omega_{p}|={\left(2W_{p}+1\right)}^{2}$ and *q* is the pixel that belongs to Ω_*p*_. The Gaussian range kernel φ (*t*) in Tomasi and Manduchi (1998) is defined as:


(2) $$\begin{array}{l} \varphi \left(t\right)=exp\;(-\frac{t^{2}}{2\sigma_{r}^{2}}), \end{array}$$

where *t* is the intensity difference between the pixels *p* and *q*. The parameter σ_*r*_ is the standard deviation of the Gaussian kernel, which determines the width of the range kernel, that is, the smoothing parameter, and σ_*r*_ is fixed at each pixel. A small σ_*r*_ gives rise to superior structure-preservation and inferior texture smoothing, and on the contrary, a large σ_*r*_ gives rise to better texture smoothing but the undesired blurring of the structure edges. Hence, it is significant to find an appropriate parameter σ_*r*_ for achieving better structure-preserving texture filtering results.

### Structure Measurement

In our proposal, we apply the large spatial kernel sizes in the homogeneous regions for texture elimination and the small sizes near structures for edge-preserving. And the kernel size at each pixel is adaptively optimized by structure measurement. So we calculate the structure information as follows.

First, we blur the input image *f* using a Gaussian filter to get image *f*_σ_. The gradient of the image *f*_σ_ is calculated by:


(3) $$\begin{array}{l} G_{p}=\sqrt{{\left(\partial_{x}f_{\sigma }\right)}_{p}^{2}+{\left(\partial_{y}f_{\sigma }\right)}_{p}^{2}}, \end{array}$$

where *G*_*p*_ is the gradient value at the pixel *p*, and ∂_*x*_*f*_σ_ and ∂_*y*_*f*_σ_ are the partial derivatives of *f*_σ_ in *x* and *y* directions.

**Figure 2:**
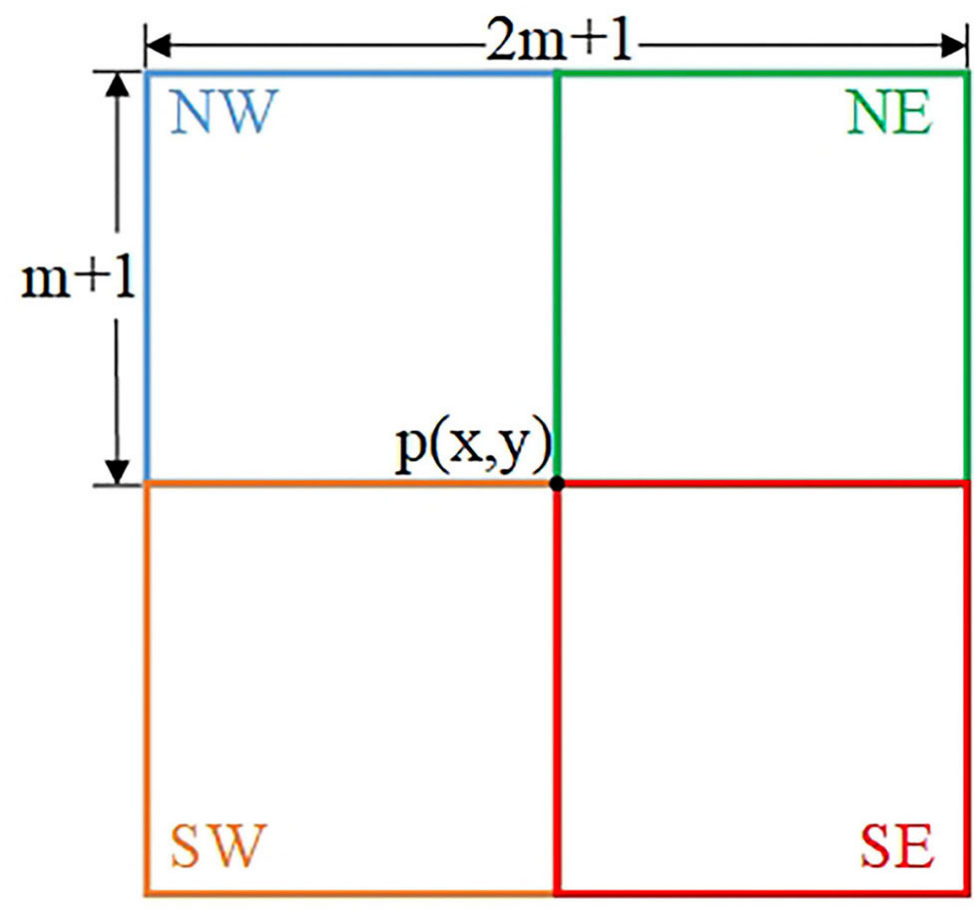
Sub-neighborhoods of four direction for structure detection.

The gradient map *G* is calculated by Equation (3), as shown in Figure 1B. It is clear that the textures with strong gradients are also preserved when only gradient information is considered, that is, the structures and textures with similar gradients cannot be completely distinguished. Therefore, in our proposal, we further conduct four-directional structure detection relying on gradient information. For each pixel, the detection neighborhood is a (2*m* + 1) × (2*m* + 1) neighborhood centered at it. To determine a more accurate structural inspection value for each pixel, we detect the (*m* + 1) × (*m* + 1) sub-neighborhoods located in four directions. Figure 2 shows the sub-neighborhoods in four directions for structure detection. To be more specific, taking into account the distance from the pixel to the exampled pixel, we computer the weighted average of the gradient values in each sub-neighborhood to obtain $A_{p}^{\left(j\right)}$, *j* = {*NW, NE, SW, SE*}, which is used to evaluate the appearance of structure edges.

In the four detecting neighborhoods of each pixel, a strong structure edge corresponds to a large *G*_*p*_ value while a weak structure edge corresponds to a small *G*_*p*_ value. For this reason, we adopt the Gaussian function as the weight to calculate the $A_{p}^{\left(j\right)}$ in the four directions, and the maximum value of $A_{p}^{\left(j\right)}$ is selected as the result of structure measurement for each pixel.


(4) $$\begin{array}{l} \{\begin{array}{c} A_{p}^{\left(j\right)}=\sum_{b\epsilon {\text{Ψ}}_{p}^{\left(i\right)}}g_{m}\left(p,b\right)\;G_{b}\\ g_{m}\;(p,b)=\frac{1}{2\pi {\left(m-1\right)}^{2}}\exp\left(-\frac{{\left\|p-b\right\|}^{2}}{2{\left(m-1\right)}^{2}}\right)\\ S_{p}=max_{j=\left\{NW,NE,SW,SE\right\}}\;\{A_{p}^{\left(j\right)}\} \end{array} \end{array}$$

where ${\text{Ψ}}_{p}^{\left(j\right)}$ represents *jth* sub-neighborhood, *b* is the pixel that belongs to ${\text{Ψ}}_{p}^{\left(j\right)}$, *g*_*m*_ (*p, b*) is the Gaussian function of the distance between the pixel *p* and *b*, and max {•} represents the maximum value of the elements in the bracket. $A_{p}^{\left(j\right)}$ is the comprehensive manifestation of the structure edges in the *jth* sub-neighborhood for the pixel *p*, whose maximum value *S*_*p*_ denotes more likelihood of the edges occurring. Therefore, the larger value of *S*_*p*_ implies less smoothing and the smaller value of *S*_*p*_ implies more smoothing around the pixel *p*.

### Adaptive Spatial Kernel Scale Estimation

From the analysis of structure measurement, a large value of *S*_*p*_ suggests that the pixel is in the vicinity of the structure edges, where the scale of the spatial kernel should be adjusted as small as possible. Conversely, the spatial kernel scale should be adjusted as large as possible in textural regions. To estimate the scale *W*_*p*_ in terms of *S*_*p*_, we establish an inverse mapping function from *S*_*p*_ to *W*_*p*_, so that the function satisfies the above conditions. The mapping can be expressed as:


(5) $$\begin{array}{l} W_{p}=\max\left\{\eta {\left(\frac{1}{\lambda }\right)}^{S_{p}^{2}},\delta \right\}, \end{array}$$

where $S_{p}^{2}$ is the square of *S*_*p*_. λ is the denominator of the base of an exponential function, whose value must be greater than 1 to ensure ${\left(1/\lambda \right)}^{S_{p}^{2}}$ ranges in [0, 1]. η is the upper limit of the scale of filtering windows, so ${\eta \left(1/\lambda \right)}^{S_{p}^{2}}$ denotes the estimated value of the spatial kernel scale. The introduction of δ is to keep the size of windows from approaching 0 so that it prevents the filtering result from over-sharpening or aliasing (δ = 1 by default). Therefore, *W*_*p*_ ranges in [ δ, η ].

### Fourier Approximation

The brute force computation of Equation (1) requires $O\left(W_{p}^{2}\right)$ operations for each pixel, which is time-consuming in practical applications, especially in textural regions, where the scale *W*_*p*_ is usually large. For the computational limitation of traditional bilateral filtering, various acceleration algorithms have been proposed to approximate the bilateral filter (Chaudhury, 2011, 2015; Chaudhury et al., 2011), whose computational complexity is decreased to *O*(1), that is, the complexity no longer depends on the scale *W*_*p*_. However, some of these algorithms cannot guarantee that the error of the approximate value of the discrete points is within the tolerance range, and the poor approximated estimation may result in color distortion in the filtering image.

In this paper, we adopt the Fourier expansion of the range kernel in Ghosh and Chaudhury (2016) to approximate the scale adaptive bilateral filter. Specifically, Equation (2) can be approximated in another manner:


(6) $$\begin{array}{l} \hat{\varphi }\;(t)=\overset{N}{\underset{n=-N}{\sum }}c_{n}exp\;(\tau nvt), \end{array}$$

where τ^2^ = −1, *v* = π/*T*, $\hat{\varphi }\left(t\right)$ is an approximate estimate of φ (*t*), *N* denotes the order of Fourier expansion, *c*_*n*_ is the corresponding coefficient, *t* is the pixel intensity differences in Ω_*p*_, and the range of *t* is {−*T*, ⋯ , 0, ⋯*T*}, where *T* can be calculated by:


(7) $$\begin{array}{l} T=\underset{p\epsilon f}{\max}\underset{q\epsilon \Omega p}{\max}|f\;(q)-f\;(p)|. \end{array}$$

For all *t* ∈ [−*T, T*], the following constraint must be satisfied:


(8) $$\begin{array}{l} |\varphi \;(t)-\hat{\varphi }\;(t)|\leq \varepsilon , \end{array}$$

where ε is the tolerance of the approximation for the Gaussian range kernel (ε = 0.01 by default).

For the given range kernel φ (*t*) and tolerance ε, the specific solution of the approximation order *N*, and the corresponding coefficients *c*_*n*_ is provided in Ghosh and Chaudhury (2016). By using Equation (6) to approximate Equation (2), we can reformulate Equation (1) as:


(9) $$\begin{array}{l} û\;(p)=\frac{E\;(p)}{H\;(p)}, \end{array}$$

where û (*p*) is an approximation of *u* (*p*), *E* (*p*) and *H* (*p*) represent the approximate value of numerator and denominator of Equation (1), respectively, which can be expressed as:


(10) $$\begin{array}{l} E\;(p)=\underset{q\in \Omega_{p}}{\sum }w\left(q-p\right)\;\hat{\varphi }\;(f\;(q)-f\;(p))f\;(q), \end{array}$$

(11) $$\begin{array}{l} H\;(p)=\underset{q\in \Omega_{p}}{\sum }w\left(q-p\right)\;\hat{\varphi }\;(f\;(q)-f\;(p)). \end{array}$$

We can further express Equations (10) and (11) as:


(12) $$\begin{array}{l} E\;(p)=\overset{N}{\underset{n=-N}{\sum }}c_{n}\exp\left(-\tau nvf\;(p)\right)\;e_{n}\;(p), \end{array}$$

(13) $$\begin{array}{l} H\;(p)=\overset{N}{\underset{n=-N}{\sum }}c_{n}\exp\left(-\tau nvf\;(p)\right)\;h_{n}\;(p), \end{array}$$

where *e*_*n*_ (*p*) and *h*_*n*_ (*p*) are expressed as follows:


(14) $$\begin{array}{l} e_{n}\;(p)=\underset{q\in \Omega_{p}}{\sum }w\;\left(q-p\right)\;f\;(q)\exp\left(\tau nvf\;(q)\right), \end{array}$$

(15) $$\begin{array}{l} h_{n}\;(p)=\underset{q\in \Omega_{p}}{\sum }w\;\left(q-p\right)\exp\left(\tau nvf\;(q)\right). \end{array}$$

Since a box function is employed for the spatial kernel, in conclusion, the adaptive bilateral filtering can be decomposed into a series of box filtering. Therefore, Equations (14) and (15) can be simplified as follows:


(16) $$\begin{array}{l} e_{n}\;(p)=\underset{q\in \Omega_{p}}{\sum }f\;(q)\exp\left(\tau nvf\;(q)\right), \end{array}$$

(17) $$\begin{array}{l} h_{n}\;(p)=\underset{q\in \Omega_{p}}{\sum }\exp\left(\tau nvf\;(q)\right). \end{array}$$

It can be added point-by-point in the neighborhood of the pixel *p* to obtain *e*_*n*_ (*p*) and *h*_*n*_ (*p*), whose computation is expensive. Hence, in our proposal, we compute Equations (16) and (17) by the recursive algorithm in Crow (1984). We assume that the integrated element of pixel *p* in *e*_*n*_ (*p*) is *r* (*q*):


(18) $$\begin{array}{l} r\;(q)=f\;(q)\;exp\;\left(\tau nvf\;(q)\right). \end{array}$$

First, we compute the integral image *R* (*p*) at the pixel *p*:


(19) $$\begin{array}{l} R\;(p)=R\;(x,y)=\overset{x}{\underset{k_{1}=1}{\sum }}\overset{y}{\underset{k_{2}=1}{\sum }}r\;(k_{1},k_{2}), \end{array}$$

where (*x, y*) is the coordinate of pixel *p* and (*k*_1_, *k*_2_) is the coordinate of the pixel in the integral region.

By using recursive theory, the integral image *R* (*x* + 1, *y* + 1) at the pixel (*x* + 1, *y* + 1) can be expressed as:


(20) $$\begin{array}{c} R\;(x+1,y+1)=r\;(x+1,y+1)\\ +R\;(x+1,y)+R\;(x,y+1)\\ -R\;(x,y). \end{array}$$

For any scale *W*_*p*_, *e*_*n*_ (*p*) can be computed as follows:


(21) $$\begin{array}{c} e_{n}\;(p)=R\;(x+W_{p},y+W_{p})-R\;(x-W_{p}-1,y+W_{p})\\ -R\;(x+W_{p},y-W_{p}-1)+R\;(x-W_{p}-1,y-W_{p}-1). \end{array}$$

Similarly, *h*_*n*_ (*p*) can be obtained.

In conclusion, we can calculate the Equations (10) and (11) according to *e*_*n*_ (*p*) and *h*_*n*_ (*p*), and instead of directly computing scale adaptive bilateral filtering, we replace each convolution with pointwise operation through Fourier expansion of the range kernel, as shown in Equations (12) and (13). Furthermore, we can compute *e*_*n*_ (*p*) and *h*_*n*_ (*p*) at *O*(1) complexity with a recursive algorithm, that is, *e*_*n*_ (*p*) and *h*_*n*_ (*p*) require a fixed number of operations for any scale *W*_*p*_.

To be specific, since Equation (21) requires three additions, this means that it takes three additions to compute both Equations (16) and (17). In summary, we can compute Equations (16) and (17) using addition operations, then we can compute Equations (12) and (13) in terms of Equations (16) and (17) by pointwise operations. It is quite clear that the computation of Equation (9) is based on Equations (12) and (13), which proves that the scale adaptive bilateral filter can be computed at *O*(1) complexity.

We compute the approximation of the output value in this paper, particularly, we consider the error to be:


(22) $$\begin{array}{l} {\left\|u-û\right\|}_{\infty }=\max\left\{|u\;(p)-û\;(p)|\;:\;p\in f\right\}, \end{array}$$

which provides the largest difference between the exact and approximate scale-adaptive bilateral filtering pixelwise.

**Table:** Necessary symbols.

**Symbol**	**Significance**
*f*	The input image
*u* (*p*)	The output value at the pixel *p*
*w* (*l*)	The spatial kernel of bilateral filtering
φ (*t*)	The range kernel of bilateral filtering
*W*_*p*_	The scale of the spatial kernel at the pixel *p*
*f*_σ_	The image blurred by input image *f*
*G*_*p*_	The gradient of the image *f*_σ_
$A_{p}^{\left(j\right)}$	The comprehensive manifestation of the structure edges
*S*_*p*_	The likelihood of the edges occurring
$\hat{\varphi }\left(t\right)$	The approximation of φ (*t*)
û (*p*)	The approximation of *u* (*p*)

According to the Equation (6), the error comes from the approximation of the range kernel, meanwhile, for all *t* ∈ [−*T, T*], $|\varphi \left(t\right)-\hat{\varphi }\left(t\right)|\leq \varepsilon$. From the conclusion of Ghosh and Chaudhury (2016), we can ensure that Equation (22) is within some tolerance:


(23) $$\begin{array}{l} {\left\|u-û\right\|}_{\infty }\leq \frac{2T\varepsilon }{w\left(0\right)-\epsilon }. \end{array}$$

Since there are complex and irregular textures in many natural images, generally, single iterative filtering cannot completely smooth out the textures. Considering this limitation, we adopt the multiple iteration operation of adaptive bilateral filtering in this paper. Algorithm 1 summarizes the overall process of our method.

**Algorithm 1:** Structure-preserving bilateral texture filtering.

**Input:** image *f*
**Output:** filtered result *u*
**for all** *i* = 1 : *I* **do**
*S* ← structure measurement *W* ← spatial kernel scale *N* ← order of Fourier expansion *E* (*p*) ← the numerator of the adaptive bilateral filter *H* (*p*) ← the denominator of the adaptive bilateral filter û (*p*) ← scale adaptive bilateral filtering of *f*
**end for**

## Experiments and Results

### Parameters Setting

Our method is implemented using MATLAB. In our algorithm, the relevant parameters are σ_*r*_, *m*, λ, η, and *I*. σ_*r*_ determines the scale of the Gaussian range kernel, as we adopt the suggested setting by Ghosh et al. (2019): σ_*r*_ ranges in [20, 40]. While evaluating structure measurement, generally, we manually set the radius of detection neighborhood *m* = 4 to practice a majority of cases. λ is used to normalize the values of structure measurement to the interval [0, 1], so we fix λ = 10 throughout.

**Figure 3:**
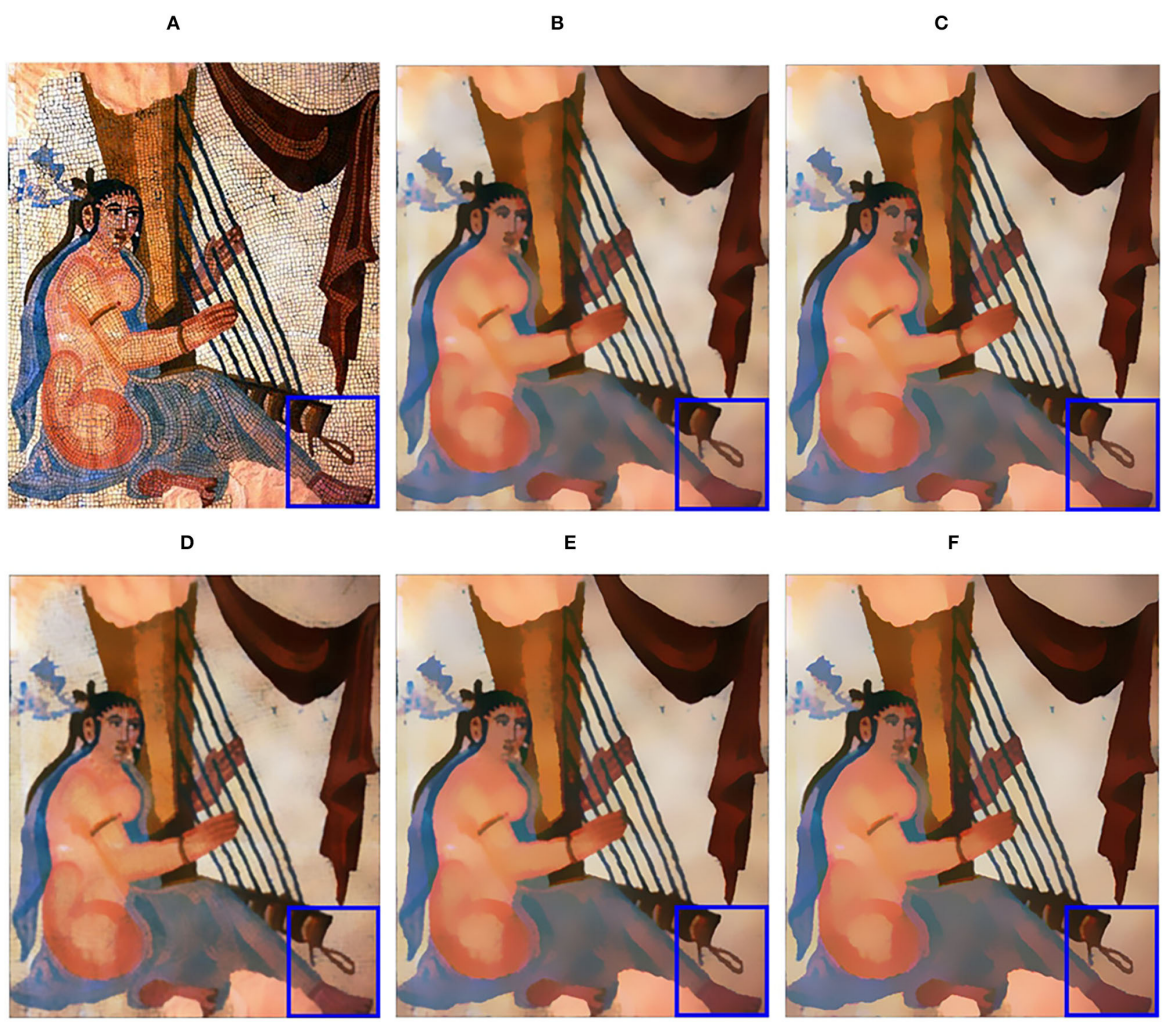
Filtering results with various parameter combinations. **(A)** Input image; **(B)** η=10,*I*=2; **(C)** η=10,*I*=3; **(D)** η=15,*I*=1; **(E)** η=15,*I*=2; **(F)** η=15,*I*=3. Reproduced with permission from Li Xu, Qiong Yan, Yang Xia, Jiaya Jia, available at http://www.cse.cuhk.edu.hk/%7eleojia/projects/texturesep/.

The relatively important parameters are the upper limit of the spatial kernel scale η and iteration number *I*. The value of η depends on the roughness of the textures and the sharpness of the structures and by the parameter recommendation of Ghosh et al. (2019), we set η ranges in [8, 16]. In most situations, setting *I* = 2 can achieve the desired filtering visual effect. Figure 3 shows the filtering results in different combinations of η and *I*.

### Visual Comparison

**Figure 4:**
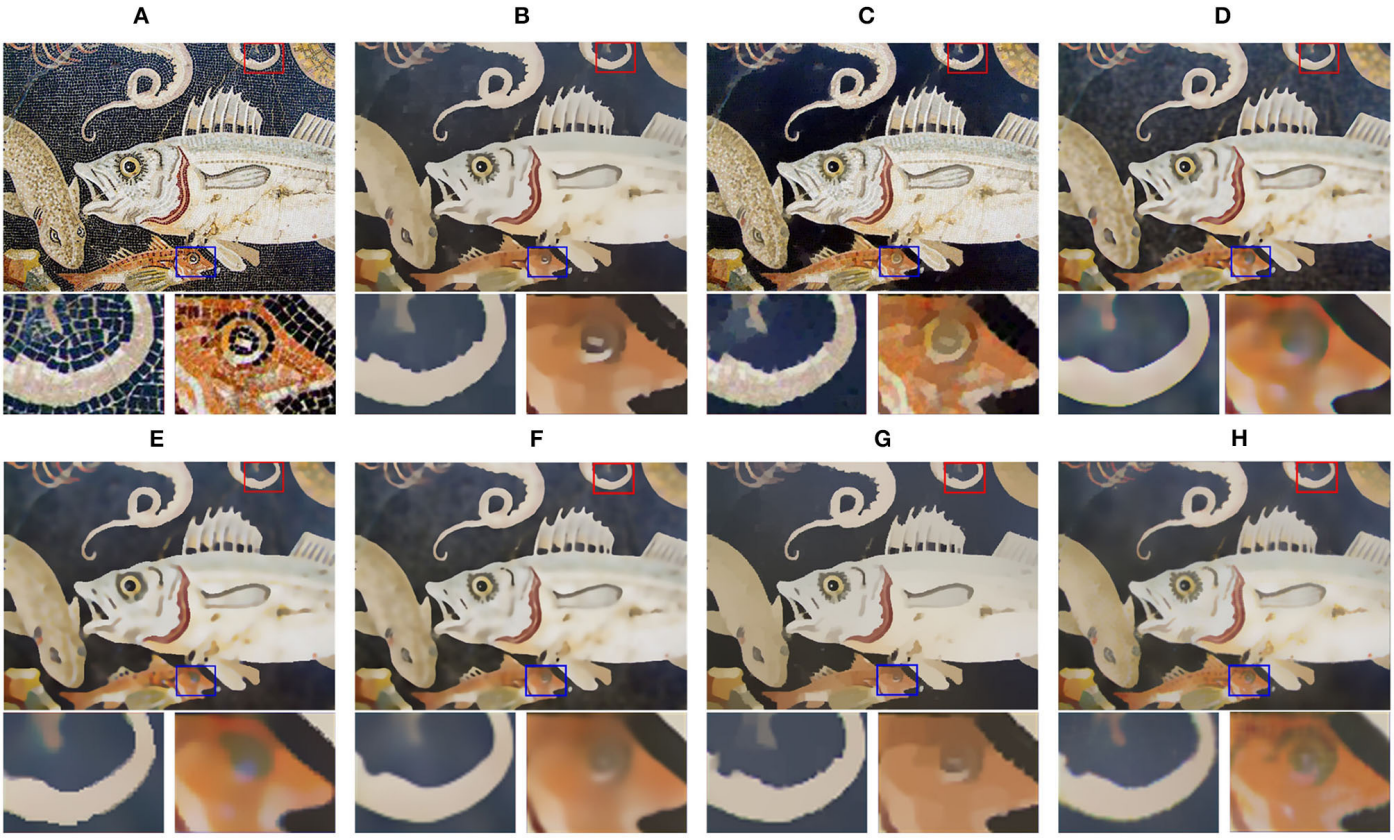
Visual effect comparison of texture filtering results. **(A)** Input image; **(B)** RTV (λ=0.015,σ=6); **(C)** SGTD (*mu*=0.31); **(D)** RGF (σ_*s*_=5,σ_*r*_=1); **(E)** BTF (*k*=9); **(F)** SATF (*sr*=0.1,*se*=0.05); **(G)** ROG (σ_1_=1,σ_2_=3); **(H)** ours (σ_*r*_=35,η=10,*I*=2). Reproduced with permission from Chengfang Song, Chunxia Xiao, Ling Lei, and Haigang Sui, available at https://sci-hub.wf/10.1111/cgf.13005.

**Figure 5:**
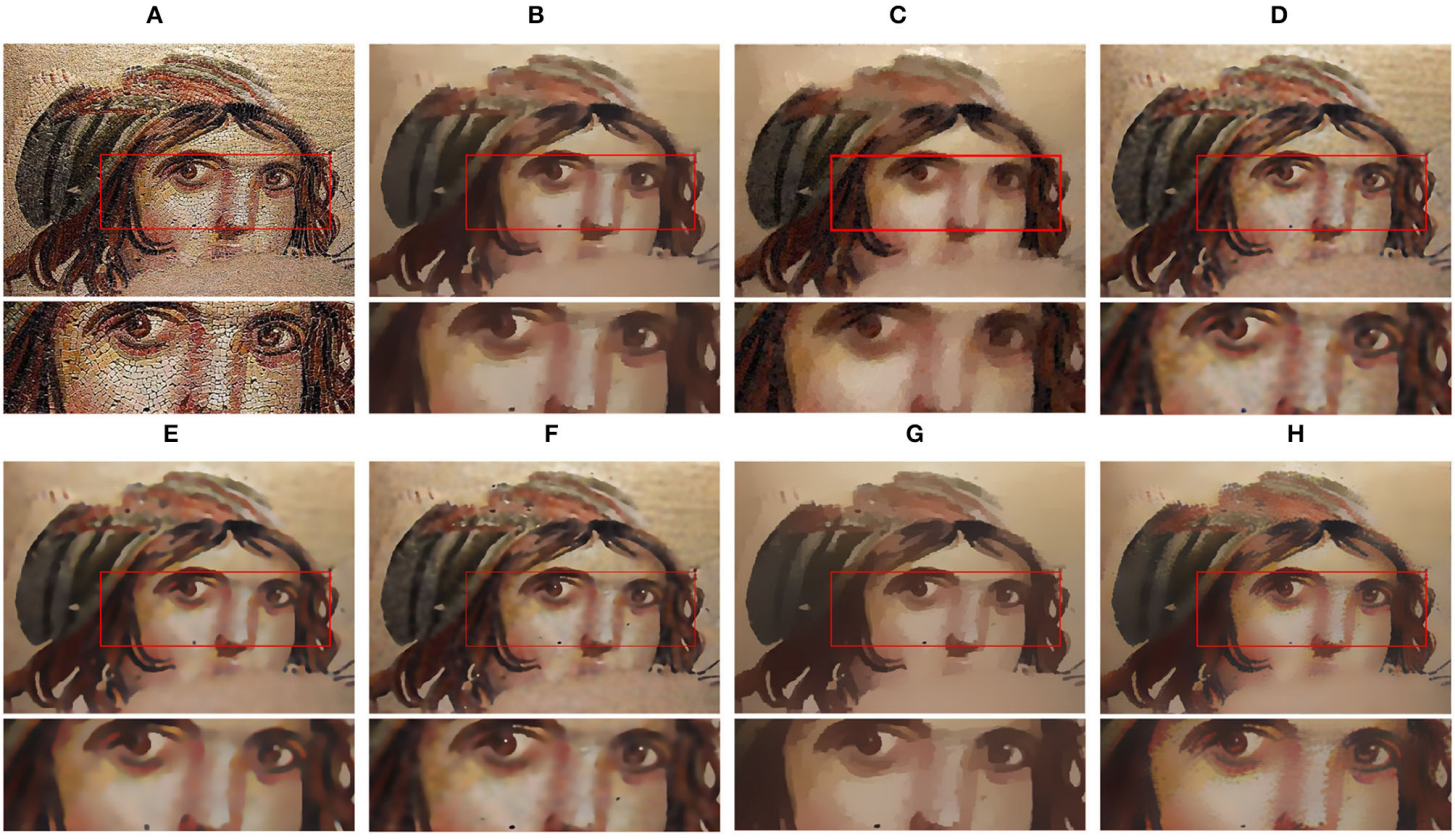
Texture filtering results comparison. **(A)** Input image; **(B)** RTV (λ=0.015,σ=8); **(C)** SGTD (*mu*=0.31); **(D)** RGF (σ_*s*_=5,σ_*r*_=0.1); **(E)** BTF (*k*=9); **(F)** SATF (*sr*=0.1,*se*=0.05); **(G)** ROG (σ_1_=1,σ_2_=4); **(H)** ours (σ_*r*_=35,η=10,*I*=2). Reproduced with permission from Sanjay Ghosh, Ruturaj G. Gavaskar, Debasisha Panda and Kunal N. Chaudhury, available at https://sci-hub.wf/10.1109/TCSVT.2019.2916589.

**Figure 6:**
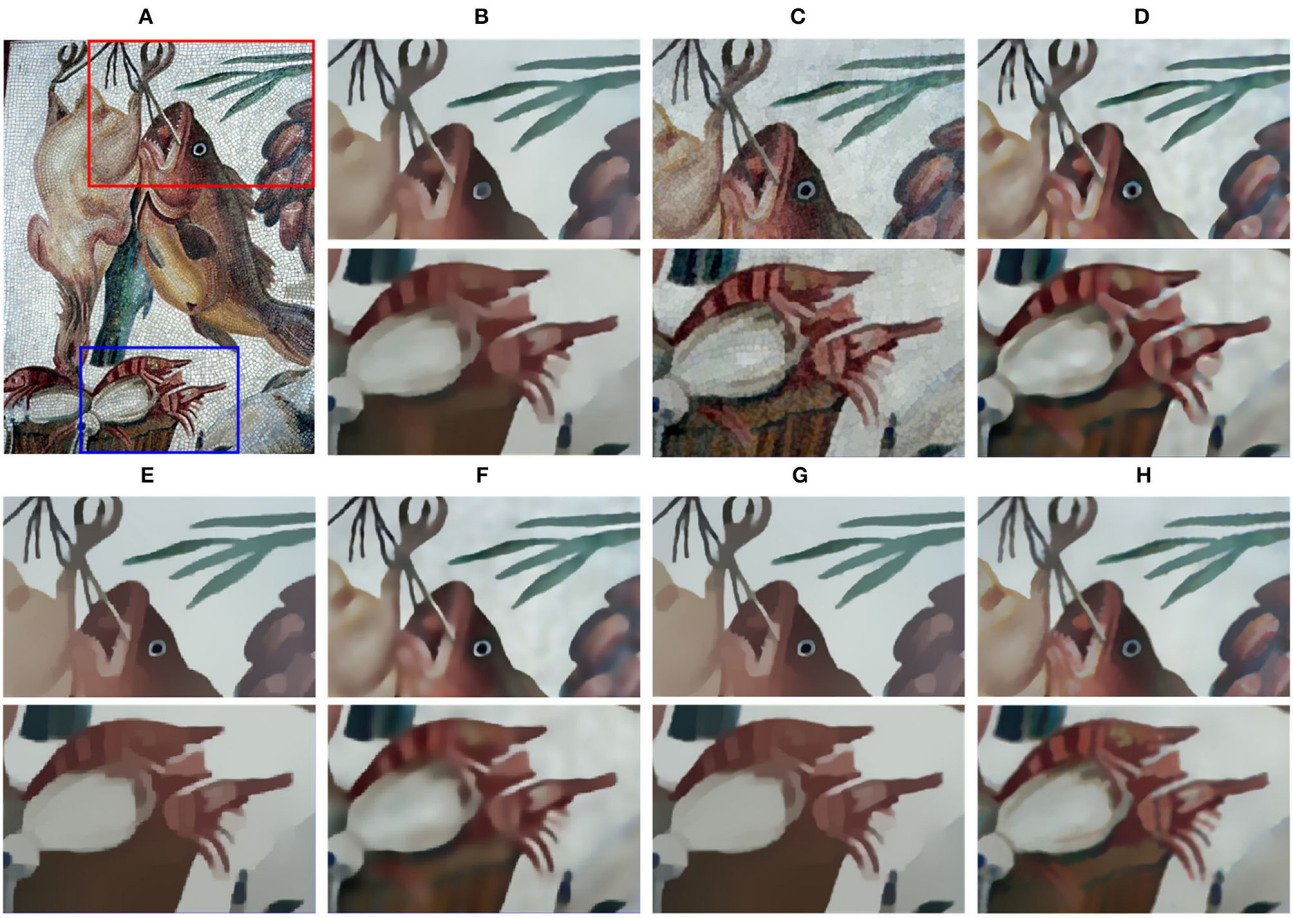
Small structures comparison of different filtering results. **(A)** Input image; **(B)** RTV (λ=0.015,σ=6); **(C)** SGTD (*mu*=0.31); **(D)** RGF (σ_*s*_=4,σ_*r*_=0.05); **(E)** BTF (*k*=9); **(F)** SATF (*sr*=0.1,*se*=0.05); **(G)** ROG (σ_1_=1,σ_2_=3); **(H)** ours (σ_*r*_=25,η=10,*I*=2). Reproduced with permission from Hyunjoon Lee, Junho Jeon, Junho Kim and Seungyong Lee, available at https://sci-hub.wf/10.1111/cgf.12875.

For subjective evaluation, we compute our algorithm with the state-of-the-art texture smoothing techniques, including relative total variation (RTV) (Xu et al., 2012), structure gradient and texture decorrelation regularization (SGTD) (Liu et al., 2013), rolling guidance filter (RGF) (Zhang et al., 2014), bilateral texture filtering (BTF) (Cho et al., 2014), scale-aware structure-preserving texture filtering (SATF) (Jeon et al., 2016), and relativity-of-Gaussian (ROG) (Cai et al., 2017). Generally, we use the suggested parameters to obtain optimal filtering results for previous methods. In Figures 4–**6**, we display the visual effect comparison for three images containing various textures and structures. The reason why we choose these three images is that they contain different types of textures and different shaped structures, which can illustrate the superiority of our algorithm from many aspects.

Figure 4 shows the filtered results of different methods on the mosaic art “Pompeii fish mosaic,” where the image contains coarse textures and highlighted small-scale structure edges. It is observed that all methods can eliminate fine details in homogenous regions; however, the approaches of SGTD and ROG perform better in removing high-contrast textures effectively. Moreover, for the preservation of small structures highlighted in the image, the methods of SGTD, RGF, BTF, and ROG can hardly preserve the fine structures of fish's eyes which are overly smoothed since the size of the windows is oversize. In the enlarged box, we can clear that the methods of RTV, SGTD, and ROG may result in excessive sharpness near structure edges, which appears as an unwanted jaggy artifact.

Compared with these existing advanced methods, our algorithm works better in eliminating coarse textures while preserving main structures as much as possible in Figure 4H. Particularly, our method can completely preserve the structure of fish's eyes.

Figure 5 shows the smoothing effect on a face image. Especially, we focus on the region highlighted with the red box, whose meaningful structures and textures on the left and right sides of the nose bridge are very similar in appearance. Since the previous methods apply texture filtering with the fix-scale kernel to remove textures, the visual effect is not always well. The results of RTV, BTF, and ROG exist unwished artifacts in the bridge of the nose. On the side, the methods of RGF and SATF perform poorly when removing high-contrast textures.

Our algorithm handles the pixel around structures with a small scale and the pixel in the textural region with a large scale. In Figure 5H, we obtain a better filtering result than the state-of-the-art methods and our method can remove coarse textures without creating artifacts.

Figure 6 shows small structures comparison of different filtering results on the mosaic art “fish.” All the existing methods blur the fine structures and cause artifacts near edges. Relatively serious are the results of RTV, SGTD, RGF, and ROG, and the whiskers and teeth of fish even became sticky. Meanwhile, in methods of BTF and SATF, the teeth of fish are barely preserved.

In contrast, our method achieves the superior property of preserving multi-scale structures, as shown in Figure 6H. The edges and details can maintain the original structure as much as possible.

**Figure 7:**
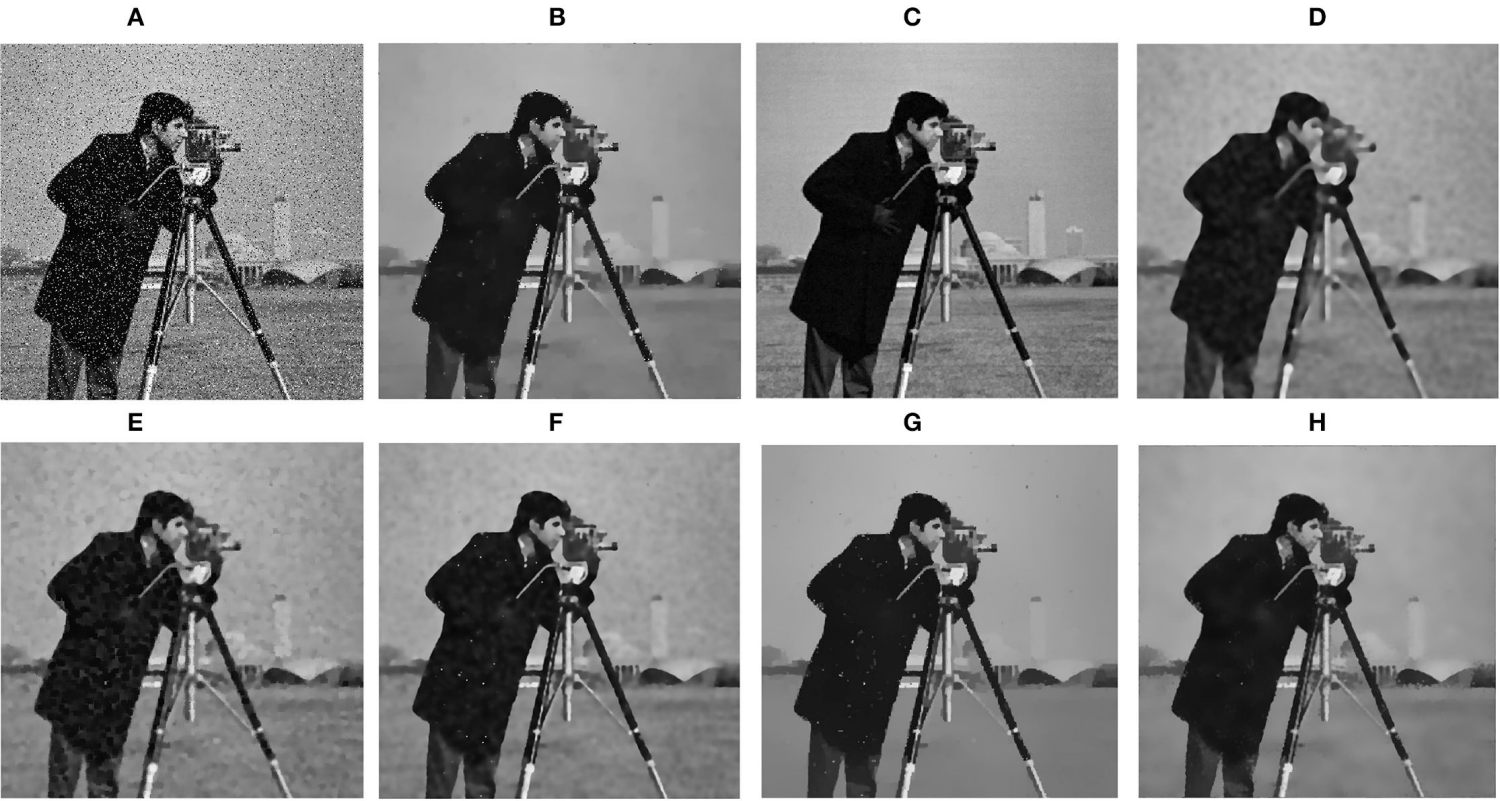
Comparison of gray image denoising. **(A)** Input image; **(B)** RTV (λ=0.015,σ=6); **(C)** SGTD (*mu*=0.31); **(D)** RGF (σ_*s*_=4,σ_*r*_=0.05); **(E)** BTF (*k*=9); **(F)** SATF (*sr*=0.1,*se*=0.05); **(G)** ROG (σ_1_=1,σ_2_=3); **(H)** ours (σ_*r*_=25,η=10,*I*=2). The picture can be found in the MATLAB public dataset, available at https://matlab.mathworks.com/.

Figure 7 shows the comparison of denoising effects on a gray image. Intuitively, it can be seen that the effects of RGF, BTF, and SATF methods are not ideal when removing gray image noise, and cannot completely smooth the noise in the background. The methods of RTV and ROG cause edge sharpening in smoothing results.

In comparison, our proposed algorithm can remove the noise of gray images and retain the edge features of people in the image, as shown in Figure 7H.

### Quantitative Evaluation

**Figure 8:**
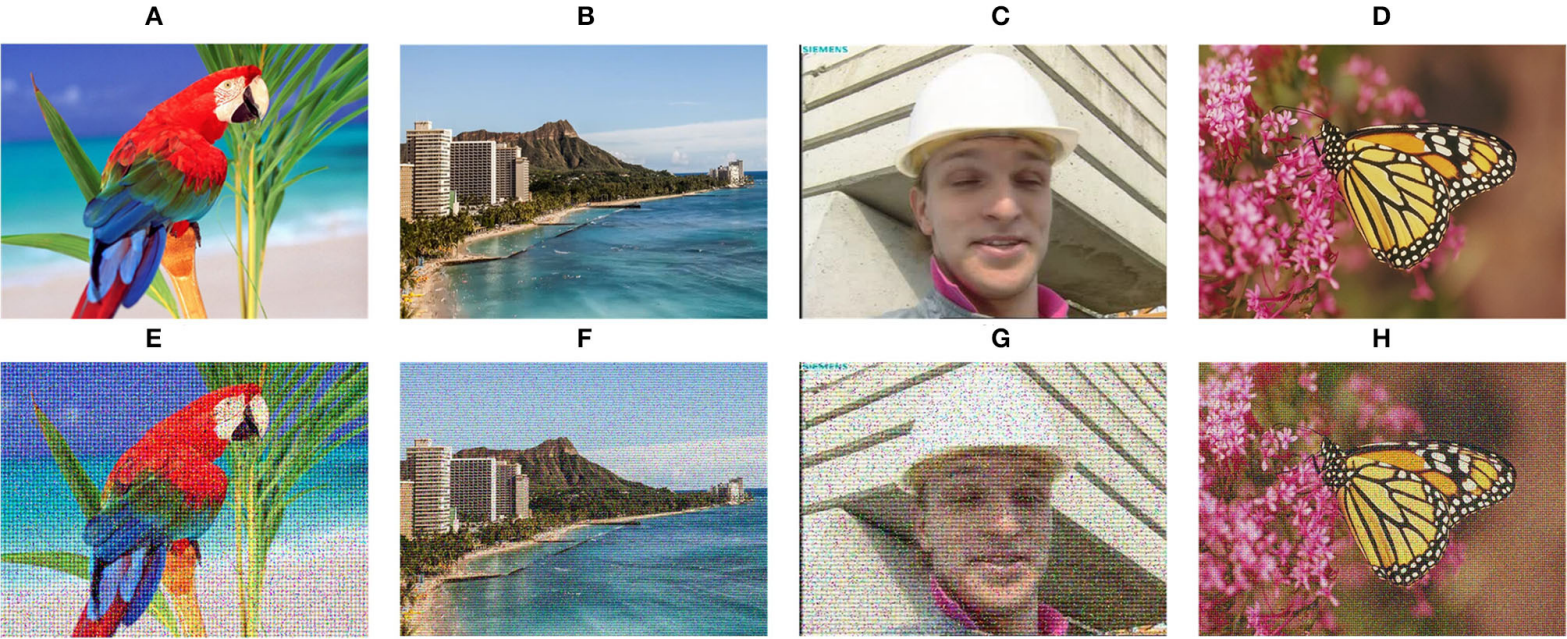
Images used for quantitative evaluation. **(A–D)** Ground truth images; **(E–H)** Images with noise. Panel **(A)** is reproduced with permission from Ryo Abiko, Masaaki Ikehara, available at https://www.jstage.jst.go.jp/article/transinf/E102.D/10/E102.D_2018EDP7437/_pdf. Panel **(B)** is reproduced with permission from Xiaoyong Shen, Chao Zhou, Li Xu and Jiaya Jia, available at http://www.cse.cuhk.edu.hk/leojia/projects/mutualstructure/. Panels **(C,D)** are reproduced with permission from Chao Dong, Chen Change Loy, Kaiming He and Xiaoou Tang, available at http://mmlab.ie.cuhk.edu.hk/projects/SRCNN.html.

The widely used image objective quantitative evaluation methods include Peak Signal-to-Noise Ratio (PSNR) and Structural Similarity Index (SSIM). PSNR is an image quality evaluation based on error sensitivity. SSIM comprehensively measures image similarity from the aspects of brightness, contrast, and structure. In our evaluation, we also take the Feature Similarity index (FSIM) (Zhang et al., 2011) and Blind Image Spatial Quality Evaluator (BRISQUE) (Chen et al., 2018) as the evaluation indexes. We selected four ground truth images in Dong et al. (2015), Abiko and Ikehara (2019), and Shen et al. (2015), and then added salt and pepper noise along with periodic noise to these four images as the texture images, as shown in Figure 8, using ground truth images as the reference to calculate PSNR, SSIM, and FSIM. In contrast, the BRISQUE is obtained only by the filtered result.

Table 1 shows the statistics of the mean values of the objective evaluation indexes of four images in Figure 8. First, on the metric of PSNR, our method performs best among these seven methods, which suggests that our results have less image distortion. The methods of RTV and SATF get higher PSNR results that are only inferior to ours. Concerning SSIM results, our method also achieves the highest result. In contrast, we only obtain the third-highest FSIM value, which is inferior to BTF and SATF. In general, the similarity between the results filtered by our approach and ground truth images is relatively good. Finally, we take a look at BRISQUE results, whose smaller score implies better perceptual quality. It just so happens that our method has the smallest BRISQUE value.

**Table 1:** Comparison of the mean values of the objective evaluation indexes.

**Methods**	**PSNR**	**SSIM**	**FSIM**	**BRISQUE**
RTV	24.1967	0.8758	0.7975	48.1249
SGTD	21.7833	0.8448	0.7773	37.5636
RGF	22.7368	0.8374	0.7946	57.4815
BTF	23.7134	0.8673	**0.8423**	43.8964
SATF	24.3114	0.8693	0.8274	55.2322
ROG	22.9508	0.8551	0.7689	49.5998
Ours	**31.3786**	**0.8799**	0.8179	**35.3667**

*The bold values indicates the maximum value of PSNR, the maximum value of SSIM, the maximum value of FSIM, and the minimum value of BRISQUE respectively*.

### Timing Data

To verify that the complexity of our algorithm does not depend on the size of the spatial kernel, we set different parameters for the upper limit of the spatial kernel scale, that is, we change the width of scale to smooth the 400 × 324 image and recode the timings required for a single iteration.

**Figure 9:**
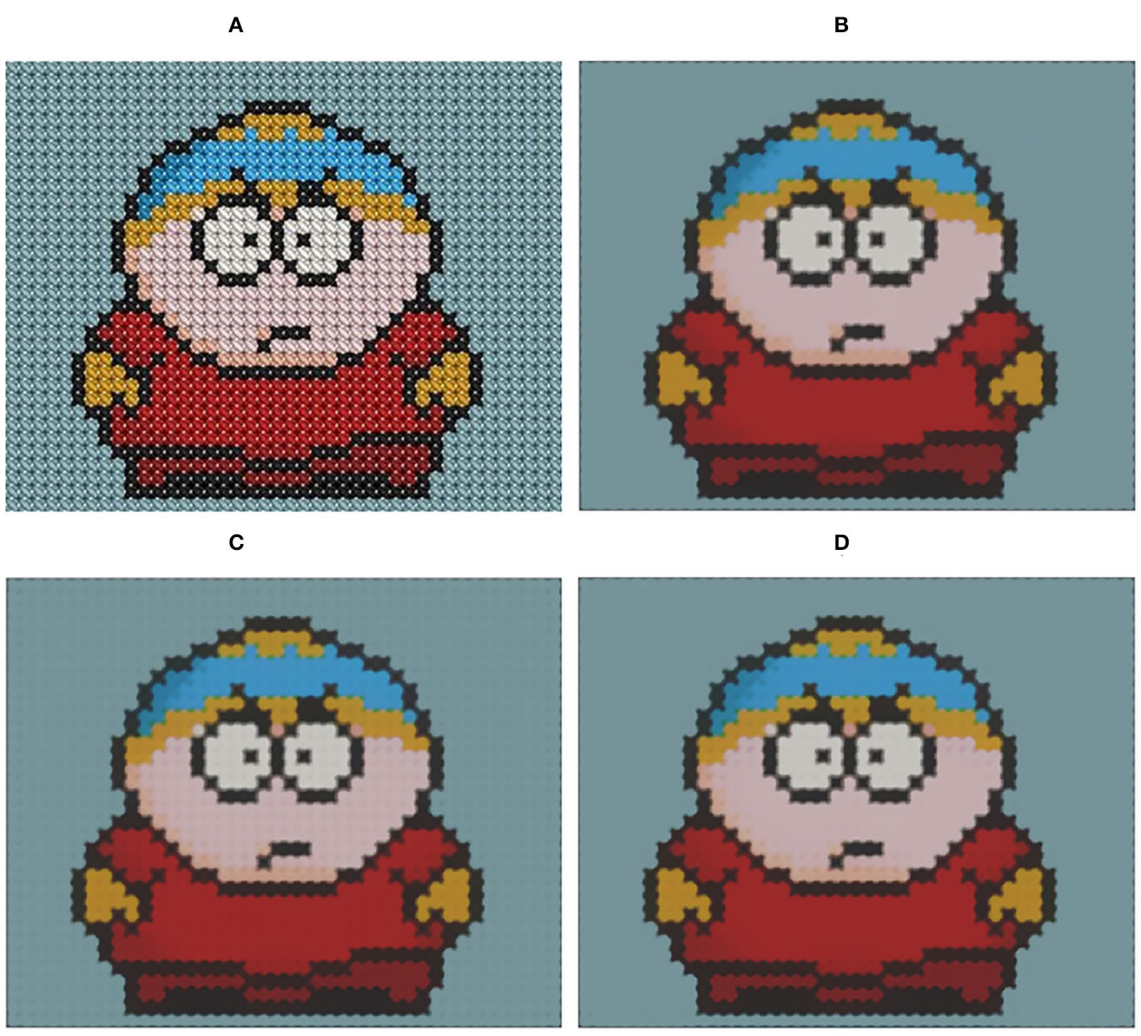
Filtered results using our method. **(A)** Input image; **(B)** (σ_*r*_=25,η=8,*I*=2); **(C)** (σ_*r*_=25,η=12,*I*=2); **(D)** (σ_*r*_=25,η=16,*I*=2). Reproduced with permission from Li Xu, Qiong Yan, Yang Xia, Jiaya Jia, available at http://www.cse.cuhk.edu.hk/%7eleojia/projects/texturesep/.

Table 2 shows the statistics of timings for a single iteration of the image in Figure 9A, Figures 9B–D show the different filtering results for different η. It can be seen that the timings required for the filtering process are not much different when the values of η are different, which illustrates that the complexity of the adaptive bilateral filtering does not depend on the size of scales. This result verifies our algorithm that the complexity is decreased to *O*(1) by our approximation of bilateral filtering.

**Table 2:** Timing statistics for a single iteration of a color image.

**Step**	**η = 8**	**η = 12**	**η = 16**
Structure map *S*	0.02s	0.01s	0.02s
Scale map *W*	0.01s	0.01s	0.01s
Texture Filtering	3.07s	3.06s	3.11s
Total	**3.10s**	**3.08s**	**3.14s**

## Applications

### Detail Enhancement

**Figure 10:**
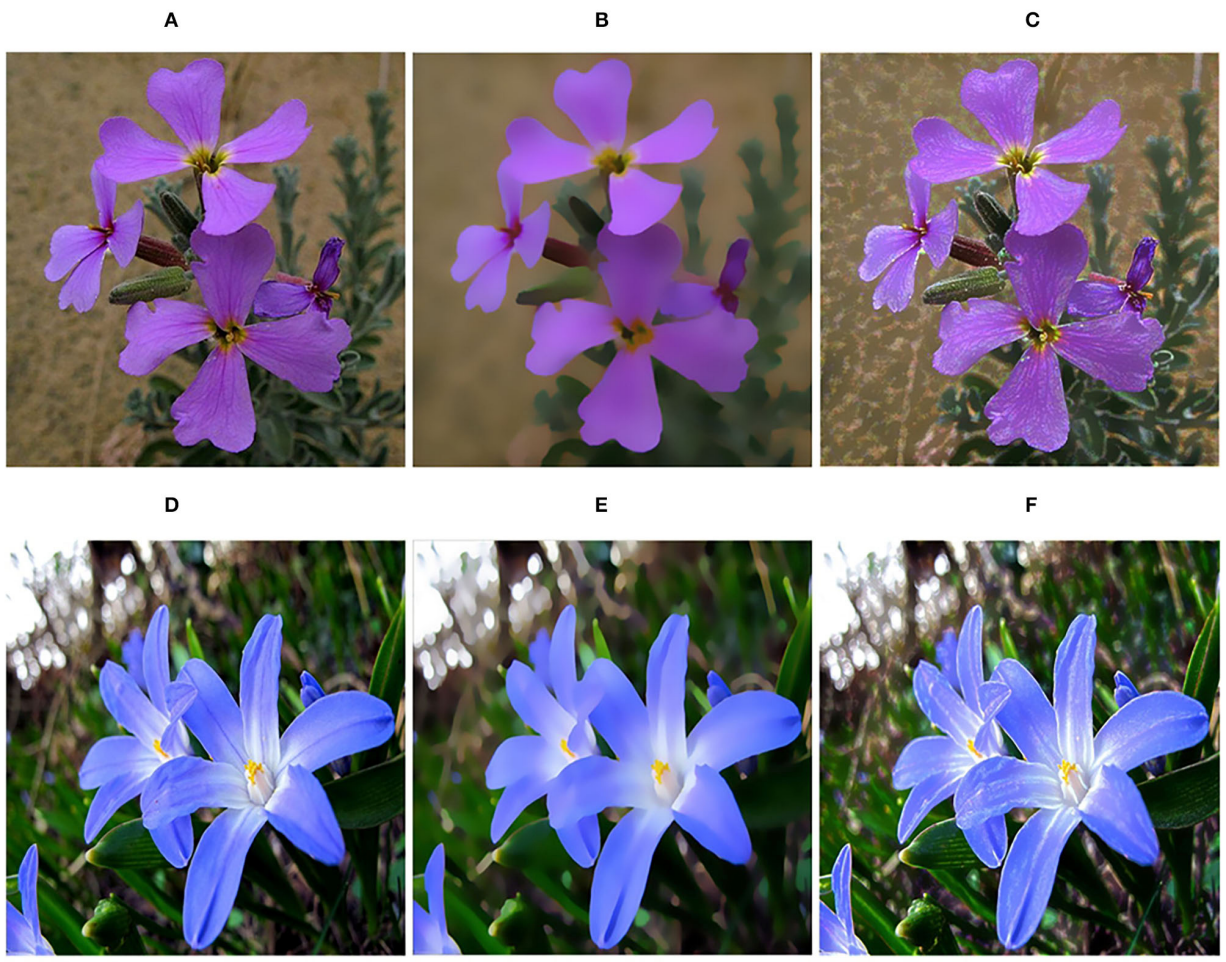
Detail enhancement. **(A,D)** Input images; **(B,E)** Filtered results using our method; **(C,F)** The detail enhancement results. Reproduced with permission from Wei Liu, Pingping Zhang, Xiaogang Chen, Chunhua Shen and Xiaolin Huang, available at https://arxiv.53yu.com/pdf/1812.07122.

Our approach can be applied to image detail enhancement (Fei et al., 2017). It aims to highlight image details and improve the visual effects of the images. Figure 10 displays the application of our method in detail enhancement. We first subtract the filtered image from the input image to generate the textures, which are magnified three times and superimposed on the input image, so that we can achieve the purpose of detail enhancement.

### Edge Detection

**Figure 11:**
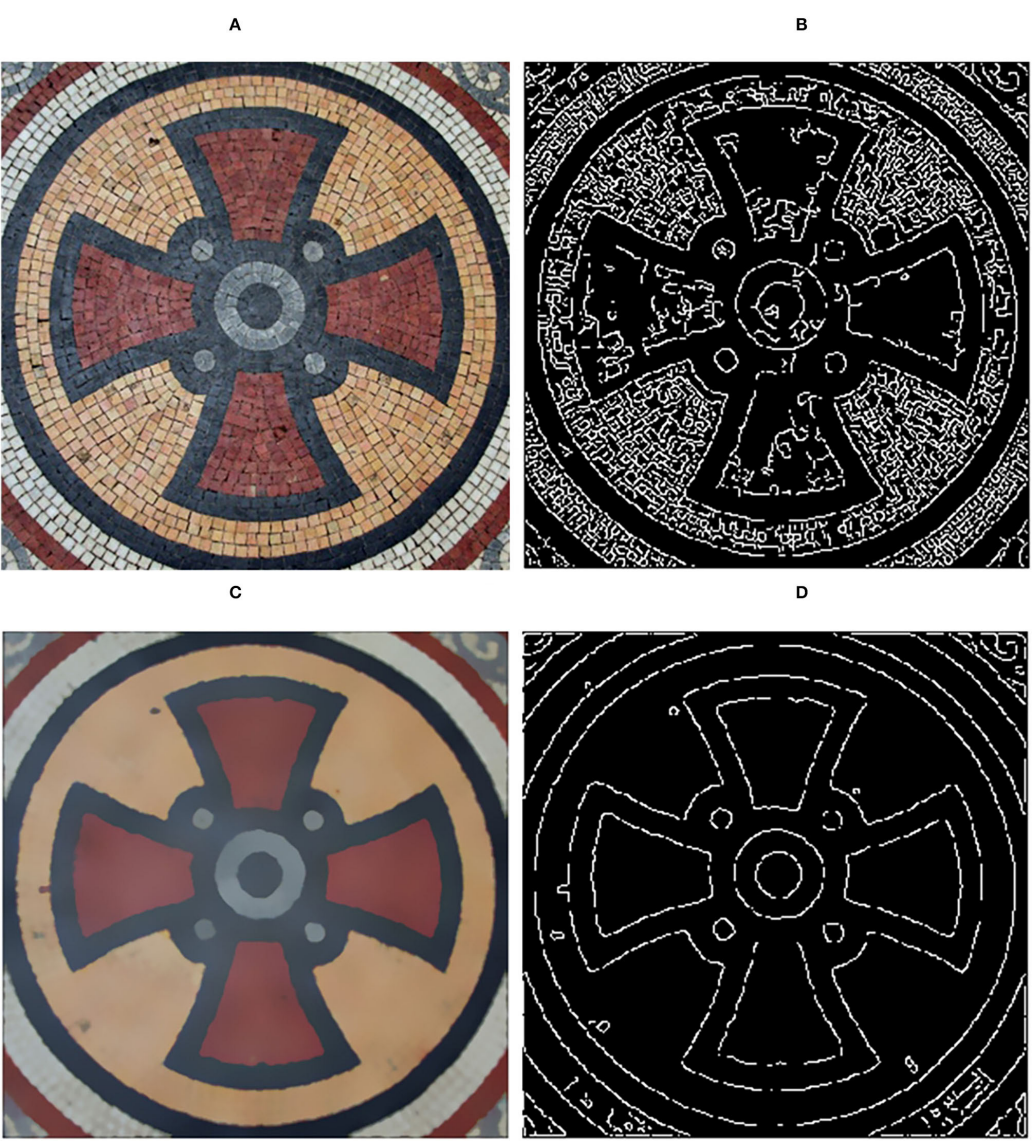
Edge detection. **(A)** Input image; **(B)** Edge detection of input image; **(C)** Filtered result using our method; **(D)** Edge detection of filtered result. Reproduced with permission from Li Xu, Qiong Yan, Yang Xia, Jiaya Jia, available at http://www.cse.cuhk.edu.hk/%7eleojia/projects/texturesep/.

The existence of high-contrast textures will keep some irrelevant information and produce false edges in edge detection. Due to the severe influence of textures, we execute our method for texture removal before edge detection. As shown in Figure 11, compared to the edge detection of the original image, the edge map of the filtered image extracted by the canny detection (Canny, 1986) operator is clearer.

### Image Abstraction and Pencil Sketching

**Figure 12:**
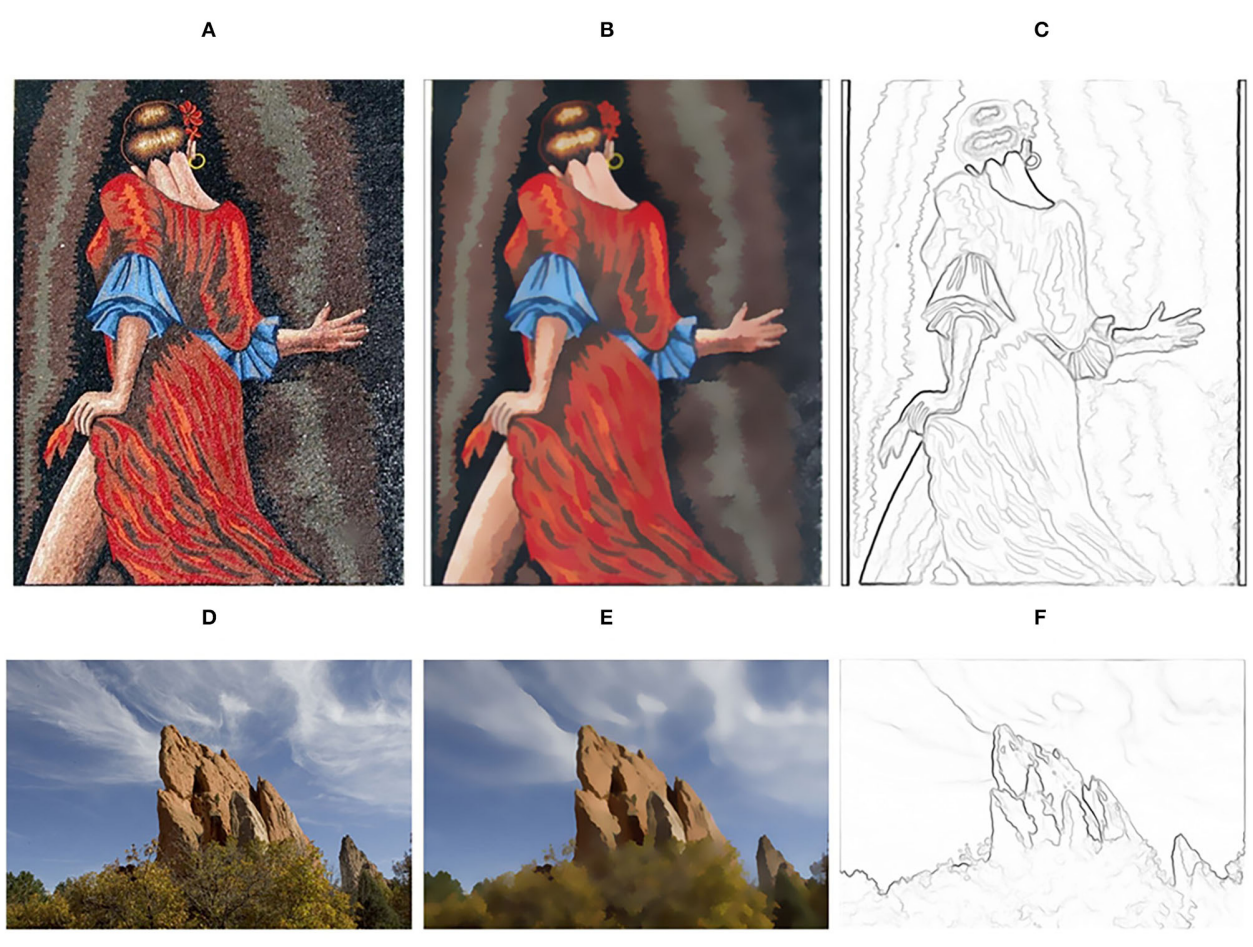
Image abstraction and Pencil sketching. **(A,D)** Input images; **(B,E)** Image abstraction results; **(C,F)** Pencil sketching results. Panel **(A)** is reproduced with permission from JiaXianYao, available at https://github.com/JiaXianYao/Bilateral-Texture-Filtering. Panel **(D)** is reproduced with permission from By Sylvain Paris, Samuel W. Hasinoff and Jan Kautz, available at https://cacm.acm.org/magazines/2015/3/183587-local-laplacian-filters/abstract.

The texture smoothing method proposed in this paper can also be applied to image abstraction and pencil sketching. Following (Winnemöller et al., 2006), our method is employed in replacing the bilateral filter to generate abstraction results. Furthermore, we obtain pencil sketching results based on image abstraction. The results are shown in Figure 12.

## Conclusion

To preserve multi-scale structures while filtering various textures, we propose an adaptive bilateral texture filter for image smoothing, whose spatial kernel scale is adjusted adaptively. To distinguish prominent structures from textures, we combine gradient information and four-direction structure inspection to generate the structure map of the image. Then, the optimal spatial kernel scale corresponding to each pixel is estimated via structure measurement, which satisfied large smoothing window sizes in texture regions and small smoothing window sizes around structures. In addition, the Fourier expansion of the range kernel is used to reduce the computational complexity. Through the subjective and objective evaluation of the experimental results, we conclude that our method performs better than existing methods in texture removal and structure preservation.

## Data Availability Statement

The original contributions presented in the study are included in the article/supplementary material, further inquiries can be directed to the corresponding author/s.

## Author Contributions

All authors listed have made a substantial, direct, and intellectual contribution to the work and approved it for publication.

## Funding

This work was supported by the National Natural Science Foundation of China (No. 62001452), the Fujian Science and Technology Innovation Laboratory for Optoelectronic Information of China (No. 2021ZZ116), and the Science and Technology Program of Quanzhou (Nos. 2020C071 and 2020C049R).

## Conflict of Interest

The authors declare that the research was conducted in the absence of any commercial or financial relationships that could be construed as a potential conflict of interest.

## Publisher's Note

All claims expressed in this article are solely those of the authors and do not necessarily represent those of their affiliated organizations, or those of the publisher, the editors and the reviewers. Any product that may be evaluated in this article, or claim that may be made by its manufacturer, is not guaranteed or endorsed by the publisher.
